# A machine learning approach to fill gaps in dendrometer data

**DOI:** 10.1007/s00468-024-02573-y

**Published:** 2024-10-15

**Authors:** Eileen Kuhl, Emanuele Ziaco, Jan Esper, Oliver Konter, Edurne Martinez del Castillo

**Affiliations:** 1 Department of Geography, Johannes Gutenberg University, Johann-Joachim-Becher Weg 32, 55128 Mainz, Germany; 2 Global Change Research Centre (CzechGlobe), Brno, Czech Republic

**Keywords:** Dendroecology, Imputation, *Acer platanoides*, *Platanus x hispanica*, Tree growth, Urban trees

## Abstract

**Key message:**

The machine learning algorithm extreme gradient boosting can be employed to address the issue of long data gaps in individual trees, without the need for additional tree-growth data or climatic variables.

**Abstract:**

The susceptibility of dendrometer devices to technical failures often makes time-series analyses challenging. Resulting data gaps decrease sample size and complicate time-series comparison and integration. Existing methods either focus on bridging smaller gaps, are dependent on data from other trees or rely on climate parameters. In this study, we test eight machine learning (ML) algorithms to fill gaps in dendrometer data of individual trees in urban and non-urban environments. Among these algorithms, extreme gradient boosting (XGB) demonstrates the best skill to bridge artificially created gaps throughout the growing seasons of individual trees. The individual tree models are suited to fill gaps up to 30 consecutive days and perform particularly well at the start and end of the growing season. The method is independent of climate input variables or dendrometer data from neighbouring trees. The varying limitations among existing approaches call for cross-comparison of multiple methods and visual control. Our findings indicate that ML is a valid approach to fill gaps in individual trees, which can be of particular importance in situations of limited inter-tree co-variance, such as in urban environments.

**Supplementary Information:**

The online version contains supplementary material available at 10.1007/s00468-024-02573-y.

## Introduction

The growth of trees on intra-annual level has been the subject of numerous studies ranging from urban tree growth (Lindén et al. 2016; Moser-Reischl et al. 2019) over experimental orchard settings (Corell et al. 2014) to forest tree analyses (King et al. 2013; Ziaco and Biondi 2018; Salomón et al. 2022; Zhang et al. 2024). Whilst undisturbed time series of dendrometer data over multiple years are desirable, many datasets contain longer periods of missing data. The primary cause of data loss is irregular physical monitoring due to the accessibility of the sites (e.g. remoteness of sites, time/cost minimization or travel restrictions during pandemics), which can result in battery power or logger failure, full data storage capacity or dendrometers at maximum. Furthermore, other technical damages like moisture intrusion, animal bites, extreme weather events (e.g. storm damage) or vandalism can result in missing values over multiple days to months. The presence of prolonged phases of missing data can impede the ability to conduct a comprehensive analysis on a given dataset, particularly when these periods coincide with the growing season, and can reduce sample size (e.g. in King et al. 2013; Corell et al. 2014; Dulamsuren et al. 2023).

To date, the most common approaches for addressing gaps in dendrometer data have been incorporated into R packages like *treenetproc* (Haeni et al. 2020; Knüsel et al. 2021), or *dendRoAnalyst* (Aryal et al. 2020). The imputation approaches are primarily based on linear or spline interpolation and are constrained to a short period of consecutive missing values (e.g. 24 measuring points) in order to achieve acceptable results (Aryal et al. 2020; Knüsel et al. 2021). In addition to these methods, Aryal et al. (2020) introduced a linear-regression-based network interpolation approach, which assumes that all individuals of a tree species at one location share similar stem growth variability. For the successful gap filling, this approach requires neighbouring trees with no missing values and high co-variance. Luković et al. (2022) tested different deep neural network architectures for gap filling and found that the combination of long short memory (LSTM) and convolutional neural networks (CNNs) performed better when an input of stem radius data and multiple climatic parameters, including temperature, relative humidity, solar radiation, and vapour pressure deficit was given. While the results of this study were promising, the authors suggested further tests of other machine learning (ML) methods and on other data.

In this study, we present a ML approach to test multiple supervised algorithms, datasets and feature combinations to reconstruct missing growth data from dendrometers. The novelty of this approach lies in its ability to fill data gaps exceeding 12 h (> 24 measuring points) of individual trees, when no supplementary data from other trees or climatic parameters are available. Furthermore, we include an evaluation of the multicollinearity of input variables and provide a straightforward scheme to be reproduced. The method was not only tested on stem growth data, but also on raw dendrometer data to expand the method to broader research applications.

## Methods

### Study location and data collection

The city of Mainz is located in western Germany (50.0° N, 8.3° E) and is defined by a temperate climate with warm summers and without a dry season (cfb, Beck et al. 2018b). The average yearly mean temperature and the average precipitation sum between 1991 and 2020 were 10.8 °C and 579.3 mm, respectively (Mainz–Lerchenberg Station: Deutscher Wetterdienst 2024).

**Fig. 1:**
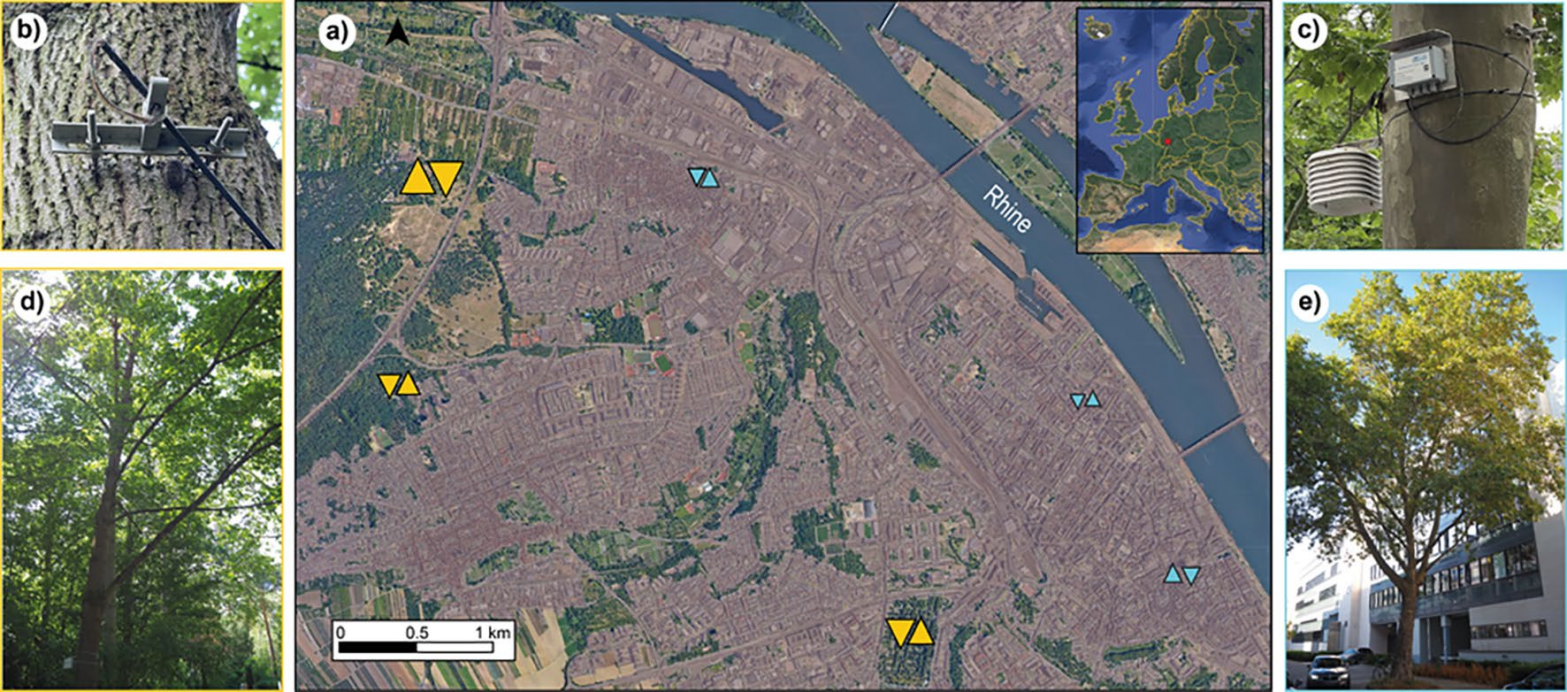
**a** Map of Mainz showing the locations in the urban (blue triangles) and non-urban (yellow triangles) areas. Triangles pointing upwards show maple trees and triangles pointing downwards show plane trees. Size of the triangles equal the size of the unsealed area around the trees relative to minimum and maximum (this figure has been prepared using European Union’s Copernicus Land Monitoring Service information; https://doi.org/10.2909/3bf542bd-eebd-4d73-b53c-a0243f2ed862 and Google Satellite Image (2024)). **b** Example of a point dendrometer. **c** Example of a full set up including a Stevenson screen. **d** Example of a non-urban maple tree. **e** Example of an urban plane tree (photo credits: D. Thimm (**b**, **c**); S. Schöfl (**d**, **e**))

Norway maple (*Acer platanoides* L.) and London plane trees (*Platanus x hispanica* Münchh.) are common urban tree species in Europe. In Mainz, these maple and plane species make up for 32.5% and 8% of the total urban tree population (Landeshauptstadt Mainz 2024 (status of 2023)). In February 2019, six maple and six plane trees were selected at different locations throughout the urban and surrounding non-urban areas of the city. On each location, point dendrometers (Ecomatik GmbH DR2), temperature and relative humidity (RH) sensors (BMC Solutions GmbH HOBO U23-001 Pro v2 data loggers) were installed (Fig. 1). All sensors measured in a 30-min interval. For this study, all measurements in the common period from April 2019 to October 2023 were used.

### Preparation of sensor and dendrometer data

First, the temperature data were subjected to quality control procedures based on the methods described in Eischeid et al. (1995), Beck et al. (2018a) and Barraro et al. (2022). The raw dendrometer data were examined using the R package *treenetproc* (Haeni et al. 2020; Knüsel et al. 2021) to identify and remove any erroneous measurements such as shifts in the data (here called jumps) related to technical failures. Here, the temperature data were used to assure no removal of frost indicated jumps. Consecutive missing values due to erroneous data in the temperature or dendrometer data, which did not exceed 24 measuring points (i.e. 12 h), were gap filled using standard linear interpolation (Haeni et al. 2020; Knüsel et al. 2021). To extract tree growth from the cleaned dendrometer data, the zero-growth model (Zweifel et al. 2016) was applied. Additional information on the trees included metadata such as the area of unsealed soil [m^2^], the diameter at breast height [cm] or the tree height [mm] (Table 1). In areas where trees were growing in a completely unsealed environment, a maximum value of unsealed area was set at 144 m^2^.

**Table 1:** Tested datasets for gap filling. *X*_*i*_ present the used features for prediction (DOY = day of year, area = unsealed area around the trees). Average diameter at breast height (DBH) and average unsealed area are given. VIF is the maximum variance inflation factor after multicollinearity tests and feature selection

#	Dataset	*X*_*i*_	Average DBH [cm]	Average unsealed area [m^2^]	VIF
1	Maple	DOY, year, hour, area	35.00	69.20	≤ 1.06
2	Plane	DOY, year, hour, area	55.22	52.14	≤ 1.06
3	Urban Maple	DOY, year, hour, area	30.37	15.24	≤ 1.06
4	Urban Plane	DOY, year, hour, area	58.67	13.22	≤ 1.06
5	Non-urban Maple	DOY, year, hour, area, tree height	39.63	123.474	≤ 1.08
6	Non-urban Plane	DOY, year, hour, area, tree height	51.77	90.75	≤ 1.08
7	6 ind. Maple datasets	DOY, year, hour	24.5 – 42.8	1.82 – 144	≤ 1.07
8	6 ind. Plane datasets	DOY, year, hour	41.5 – 88.7	2.77 – 144	≤ 1.07

To find the optimal approach for gap filling, a series of tests was conducted utilising a combination of different datasets (Table 1) and methods (Table S1). Two hourly-resolved datasets for each species, maple and plane, were built including measurements from all locations (datasets #1–2). Four additional datasets were constructed by splitting the species-specific ones into urban and non-urban locations per species (datasets #3–6). Furthermore, these datasets were utilized to investigate the effects of data size and hyperparameter tuning on the model performances. Hyperparameter tuning enables data scientists to adjust model hyperparameters for optimal performance, thereby avoiding overfitting to the training data (Géron 2019). Additionally, 12 datasets representing the individual trees were subject to testing (summarized in #7–8). The presence of multicollinearity among predictive variables was evaluated using a variance inflation factor threshold of 5 (Dormann et al. 2013). Correlated variables were excluded, leaving a specific set of predictors for each dataset (hereafter called features *X*_*i*_): day of the year (DOY), year, hour, and area (set as constant value per tree). In the non-urban datasets, tree height was included as predictor, as VIF values were below five. All mentioned data processing steps were computed in R 4.2.2 (R Core Team 2021).

### Machine learning implementation

First, the outputs from the zero-growth models were controlled for incorrect values, namely negative values or growth at the end (DOY > 304) or start (DOY < 60) of each year. Manual data quality control is required to detect incorrect growth values or artefacts in winter, as bark cell degradation is not taken into account in the zero-growth model (Zweifel et al. 2016). Matrices comprising the features *X*_*i*,_ and the growth labels *y* were built and rows with missing dendrometer data were excluded during model training process. Subsequently, *X* and *y* were split into *training* (80%) and *test* (20%) subsets using stratified sampling of the features ‘year’ and ‘hour’ to ensure that the distribution of data in the subsets is balanced. Afterwards, all features were normalized using z-transformation. The parameters of the z-transformation of the *training* subset were employed to normalize the *test* subset data and the input data in periods of missing values.

To find the best model, datasets #1–6 were fitted to eight supervised ML algorithms for regression problems and tested though repeated 10-fold cross-validation (10 repeats) on the *training* subsets before hyperparameter tuning (see Table S1 for a list of the algorithms). To evaluate the performance of these ML regression models, the root mean squared error (RMSE) and the adjusted *R*^2^ were calculated. Significant differences in the performances of the algorithms were checked with the Friedmans test (Rainio et al. 2024). RMSE results between two models were analysed using the Mann–Whitney U test. The best performing algorithms were hyperparameter tuned using a 10-fold cross-validation and Bayesian Optimization Search (iterations = 70) (Bischl et al. 2023). Afterwards, tuned models were evaluated on the *test* subsets. The effects of the hyperparameter tuning and its necessity for gap filling were analysed. Permutation feature importance (PFI) was used to analyse the features and their predictive power by calibrating and validating the model for each permutation (boot = 50). The PFI value is here defined as the mean difference between the original and the permuted *R*^2^ of the model (Breiman 2001; Schwarz et al. 2024). A ‘random’ feature was included to provide a statistical baseline for random performance decline.

**Fig. 2:**
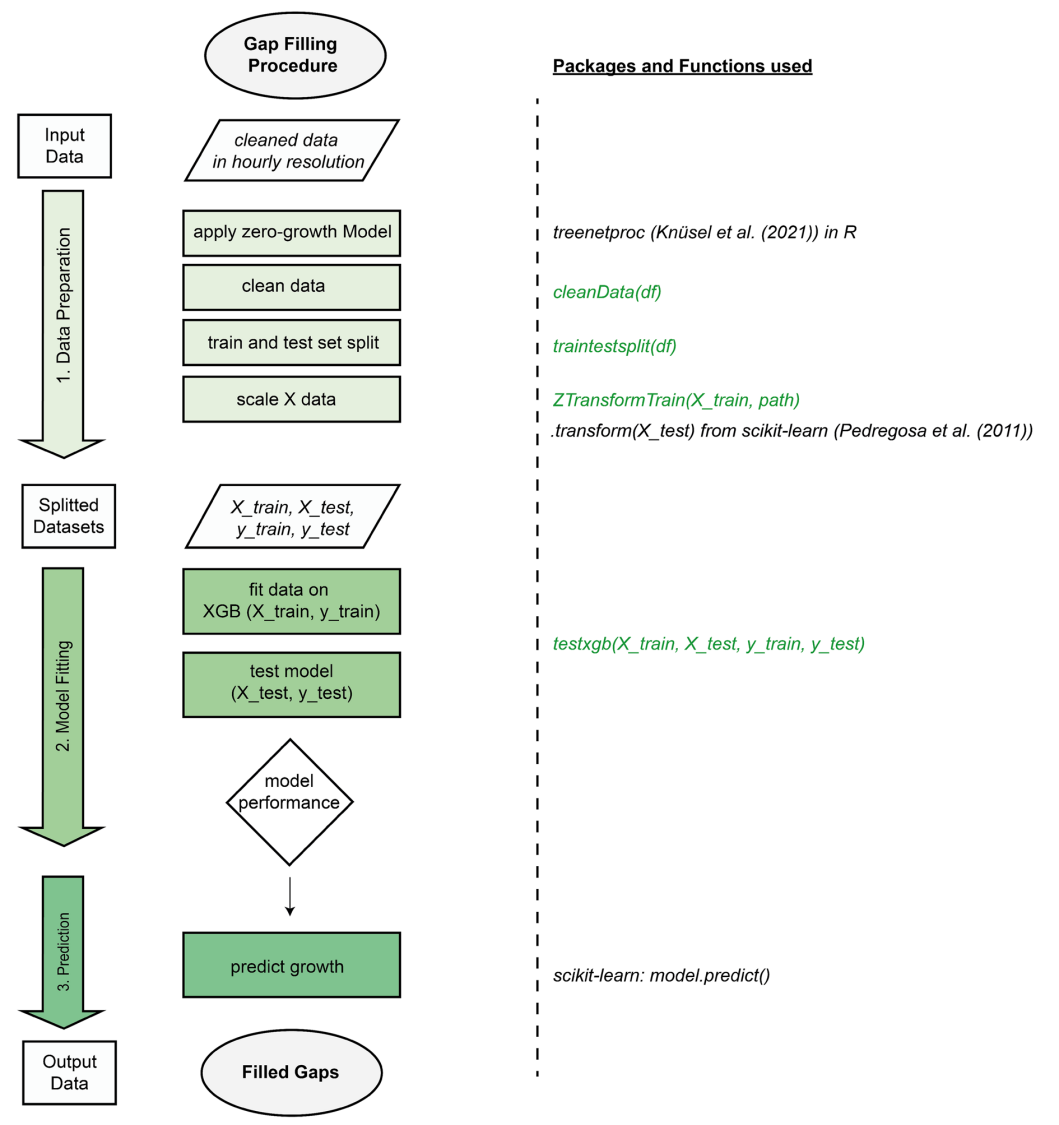
Gap filling process scheme using extreme gradient boosting to fill long data gaps and needed functions and packages. Green functions are implemented for this study and are available on GitHub

A process scheme was developed following the necessary steps and functions for gap filling dendrometer data (Fig. 2). The functions created for this project, along with detailed explanations and data to replicate the process, are available at GitHub (https://github.com/ESKuhl/DM_GF_XGB). The functions are based on the *scikit-learn* package (Pedregosa et al. 2011) and are intended to simplify the application. The initial stage of the process is the data preparation, during which the zero-growth model is applied (Zweifel et al. 2016; Haeni et al. 2020; Knüsel et al. 2021) and corrected for erroneous values in the winter months. A dataset associated with the growth data must be constructed with continuous values in the features ‘DOY’, ‘year’ and ‘hour’. For the functions to work properly, a variable called ‘Label’ must be added, which includes the output of the zero-growth model (i.e. growth labels *y*) with data gaps. Once the data have been prepared, they are fitted to an ML algorithm in the second step using the function testxgb(), which returns the performance results and the *model*. If the model performance on the *test* subset indicates a high degree of accuracy, the gaps in the dendrometer data can be filled in the third step using the *model*.predict() function of *scikit-learn*.

### Gap filling model performance

To assess the functionality of this approach across different phases of the growing season, artificial gaps of 30 consecutive days were created on datasets #3–8 for the start (April 16^th^–May 15^th^), middle (June 1^st^–30^th^) and end (September 1^st^–30^th^) of the growing season for each year of observation (2019–2023). The resulting 15 subsets, comprising the three-period/5-years combinations for each original dataset, were individually fitted to the ML algorithms and tested, yielding 240 models for comparison. Moreover, to benchmark the new approach against existing methods, network interpolation from Aryal et al. (2020) and spline interpolation were applied to the same artificial gaps, if the data allowed for it.

All model fittings, runs and analyses were conducted with Python 3.9.19 and the packages *scikit-learn* (Pedregosa et al. 2011), *scikit-optimize* (Head et al. 2018) and *xgboost* (Chen and Guestrin 2016). The analysis can be replicated in R by utilizing the package *reticulate* (Ushey et al. 2024), which enables the execution of Python functions in R studio when Python and the necessary packages are installed on the device (for further details see: https://rstudio.github.io/reticulate/).

## Results and discussion

### Algorithm selection and model evaluation

The eight algorithms significantly differed in their performances across all datasets (**Table S2** for datasets #3–6). In all runs, nonlinear algorithms outperformed linear models and exhibited higher adjusted *R*^2^ and lower RMSE mean values. The three best performing untuned algorithms were identified as random forest (RF, Breiman 2001), extreme gradient boosting (XGB, Chen and Guestrin 2016) and k-nearest-neighbour (kNN, Fix and Hodges 1951). Although the linear models performed significantly worse relative to the nonlinear ones, RMSE values did not differ significantly between each other. We selected the best three algorithms (RF, XGB and kNN) for further hyperparameter tuning. Ridge regression (Hoerl and Kennard 2000) was additionally included as fourth algorithm to assess the impact of hyperparameter tuning on a linear model.

After hyperparameter tuning, the RMSE values for kNN and XGB showed either minimal improvement or no change (see **Table S3** for datasets #3–6). The RMSE values for RF increased marginally after tuning from 0.00 to a maximum RMSE of 0.07. Ridge regression RMSE values ranged between 0.56 and 0.96 and could not be improved by tuning. Model performances before and after the tunings revealed no significant differences. The species-specific models showed similar results. The results of the *test *subsets of these models (**Table S4**) demonstrated that the models exhibit comparable performances on the *test* subset data and on the *training* subset data after tuning. When the algorithms were fitted to individual tree data without hyperparameter tuning, the results showed similarly low RMSE values.

The computation time required for hyperparameter tuning is substantially growing with the dataset size. While the time to fit the data without hyperparameter tuning has a maximum of a few minutes, hyperparameter tuning can take from 20 min (individual small datasets, approx. 25,000 datapoints) to 8 h (model #3–6, approx. 90,500 data points) and exceeds 24 h when the species-specific datasets are tuned (180,000 data points). Due to the increased computation time, which did not significantly improve model performances, the suggested procedure (Fig. 2) did not include hyperparameter tuning.

**Fig. 3:**
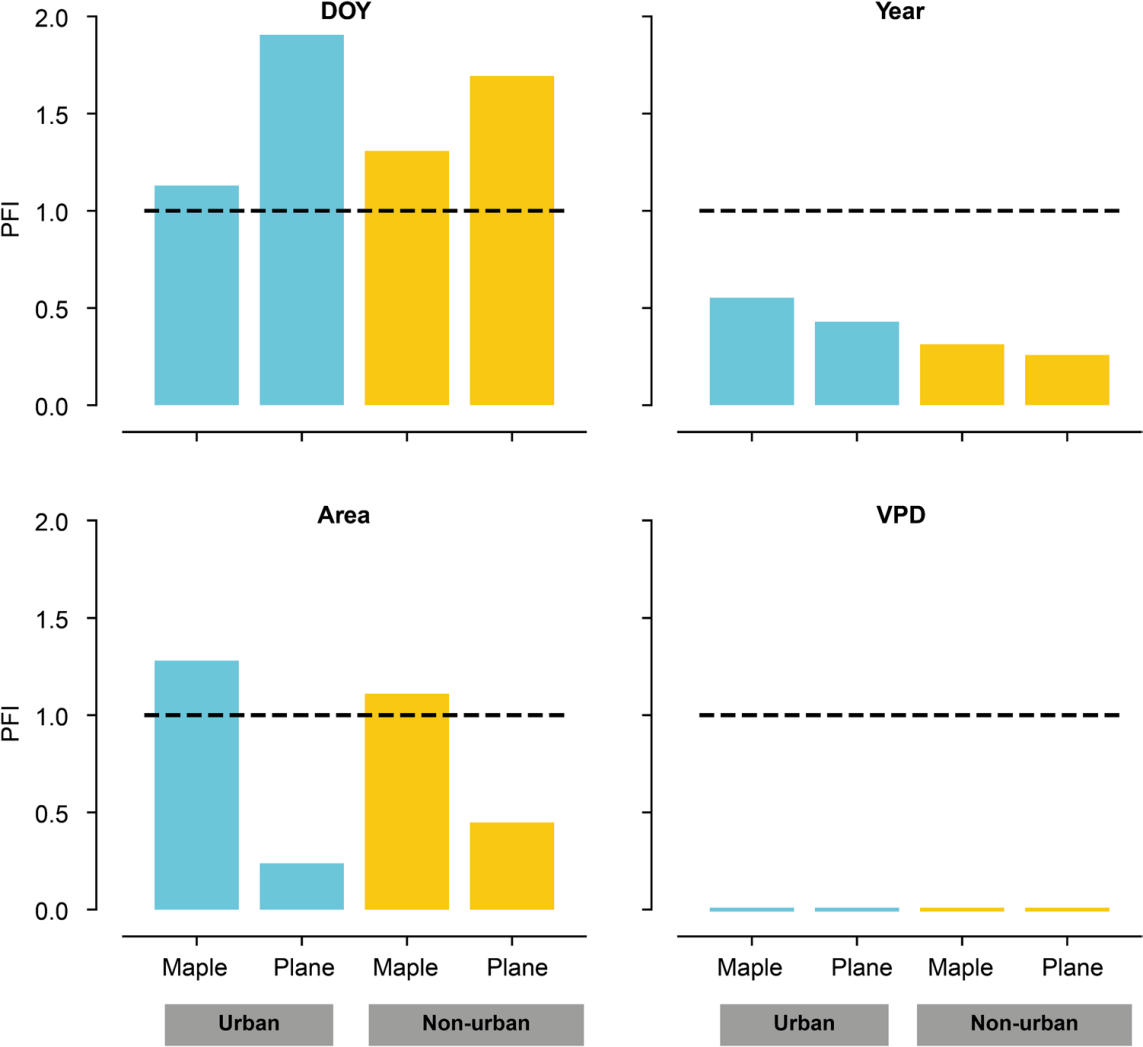
Example of permutation feature importance (PFI) results for the three most important features DOY (day of year), year and area of the models derived from the urban (blue) and non-urban (yellow) multi tree datasets #3–6 (Table 1). The figure contains an additional example of the PFI values from climatic features like the vapour pressure deficit (VPD), when these are included in the models

Among the predictor variables included in the datasets, the day of the year (‘DOY’) was the most important feature of the grouped tree models (Fig. 3). The PFI values exceeded 1 for the ‘DOY’ variable in all grouped tree models, with the highest values observed in the plane tree models. This indicates that the permuted ‘DOY’ models are poorly fitting with negative *R*^2^ values. In the maple tree-growth models, the feature ‘area’ achieved a higher ranking than in the plane-tree-based models. The feature ‘year’ also had a significant predictive value for all four algorithms. Despite this, an increase in model error was not significantly observed for the features’tree height’ or ‘hour’, as their PFI values were not exceeding the PFI values from the introduced random feature. Additional tests including various climatic parameters revealed no significant PFI values for these parameters, which is example wise shown for VPD in Fig. 3 and demonstrates that these variables are not essential for gap reconstruction using this ML approach.

All four algorithms were trained as individual and grouped tree models on the datasets with the consecutive gaps at the start, middle and end of the growing seasons. No hyperparameter tuning was applied. The average performance of these models on their *test* subsets highlights the superiority of the individual tree models (**Table S5**). The comparison between the four algorithms clearly showed that in most of the cases, the decision-tree-based algorithms outperformed kNN and ridge regression. The performance of ridge regression and kNN was found to be significantly different to XGB and RF, respectively (*p* < 0.01), with XGB and RF being similar in their performance (*p* > 0.1). All individual tree models performed on average similarly good than on their *training* data. The grouped tree models, on the other hand, had higher average RMSE values on the *test* subset data compared to the *training* subset data. This is an indicator for generalization errors in the grouped tree models. Although these models performed well on the *test* data with hyperparameter tuning (**Table S4**), it is probable that the grouped tree models are dependent on the tuning to prevent overfitting on the *training* subset data. The diverse growth patterns of multiple trees might have forced the models to learn detailed patterns, which are characteristically for the *training* data, but are too specific for a general application to unseen data (Géron 2019).

As RF and XGB showed similar performance on the *test* subsets, a Mann–Whitney *U* test validated that the RMSE values of the artificial gap predictions were not significant different between the algorithms (*p* > 0.01) (Fig. S1). These results indicate that XGB is the optimal algorithm for this gap-filling approach. The algorithm is known for its high scalability, short computation time, the ability to handle unbalanced datasets and its iterative learning process (Chen and Guestrin 2016; Fatima et al. 2023). Compared to randomly build decision trees in RF, XGB grounds on a gradient boosting algorithm (Friedman 2001) and iteratively builds an ensemble model of decision trees. The objective of each iteration is to minimize the loss function. The model optimization stops, when the performance of the *training* or the *validation* subset, a 20% subset from the *training* subset, no longer improves. Consequently, overfitting can be mitigated without the need for hyperparameter tuning (Ying 2019).

### Evaluation on artificial gaps

**Fig. 4:**
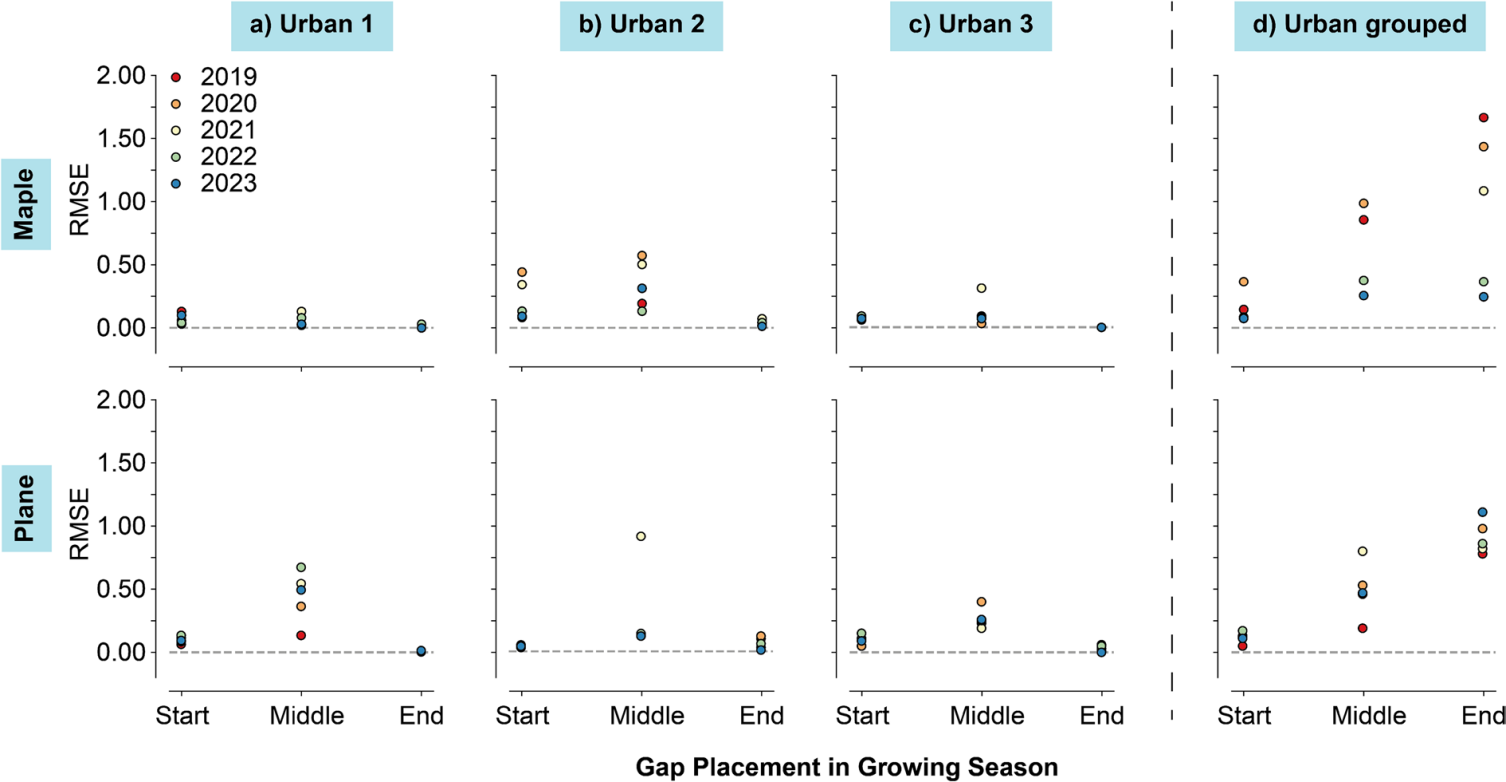
RMSE results for the predictions on all different phases (Start, Middle and End of growing season) and all years (2019–2023) for the urban individual (**a–c)**) grouped tree models (**d)**)

**Fig. 5:**
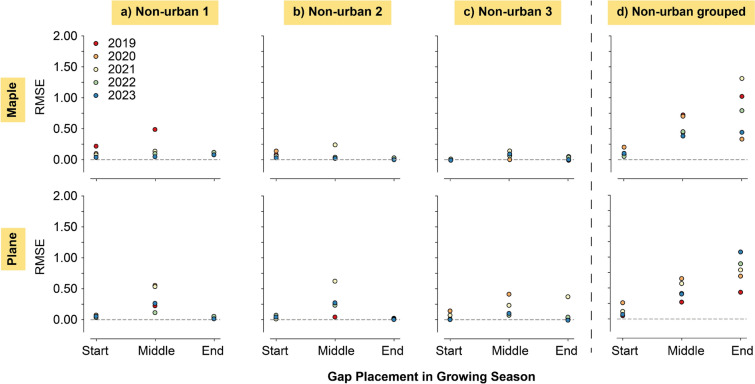
RMSE results for the predictions on all different phases (Start, Middle and End of growing season) and all years (2019–2023) for the non-urban individual (**a–c)**) grouped tree models (**d)**)

Both individual and grouped tree models predicted data for the artificially created growth gaps to evaluate the performances on consecutive missing values. RMSE values for the various gaps were calculated using the excluded observations (Figs. 4 and 5). The values for the grouped tree models in Figs. 4d and 5d were generally higher than for the individual tree models in a–c. Despite the performances of the grouped models, gap filling in the middle of the growing season showed higher variance in RMSE values than at the start and middle for the individual tree models. Best predictions of the individual tree models were observed for the gaps at the end of the growing season. In this period, the performances in the grouped models were worse compared to all other periods. It is likely, that XGB was unable to predict growth with the same degree of accuracy when multiple trees with differing growth behaviours and diverse growing season lengths were given as *training* data. From the grouped tree models, the urban models had higher RMSE values than the non-urban models, which could indicate more coherent tree-growth patterns between non-urban trees. Urban environments have shown high heterogeneity of tree-growth influencing conditions between urban locations and compared to non-urban environments (Iakovoglou et al. 2001; Cedro and Nowak 2006; Moser-Reischl et al. 2019; Lv et al. 2024).

**Fig. 6:**
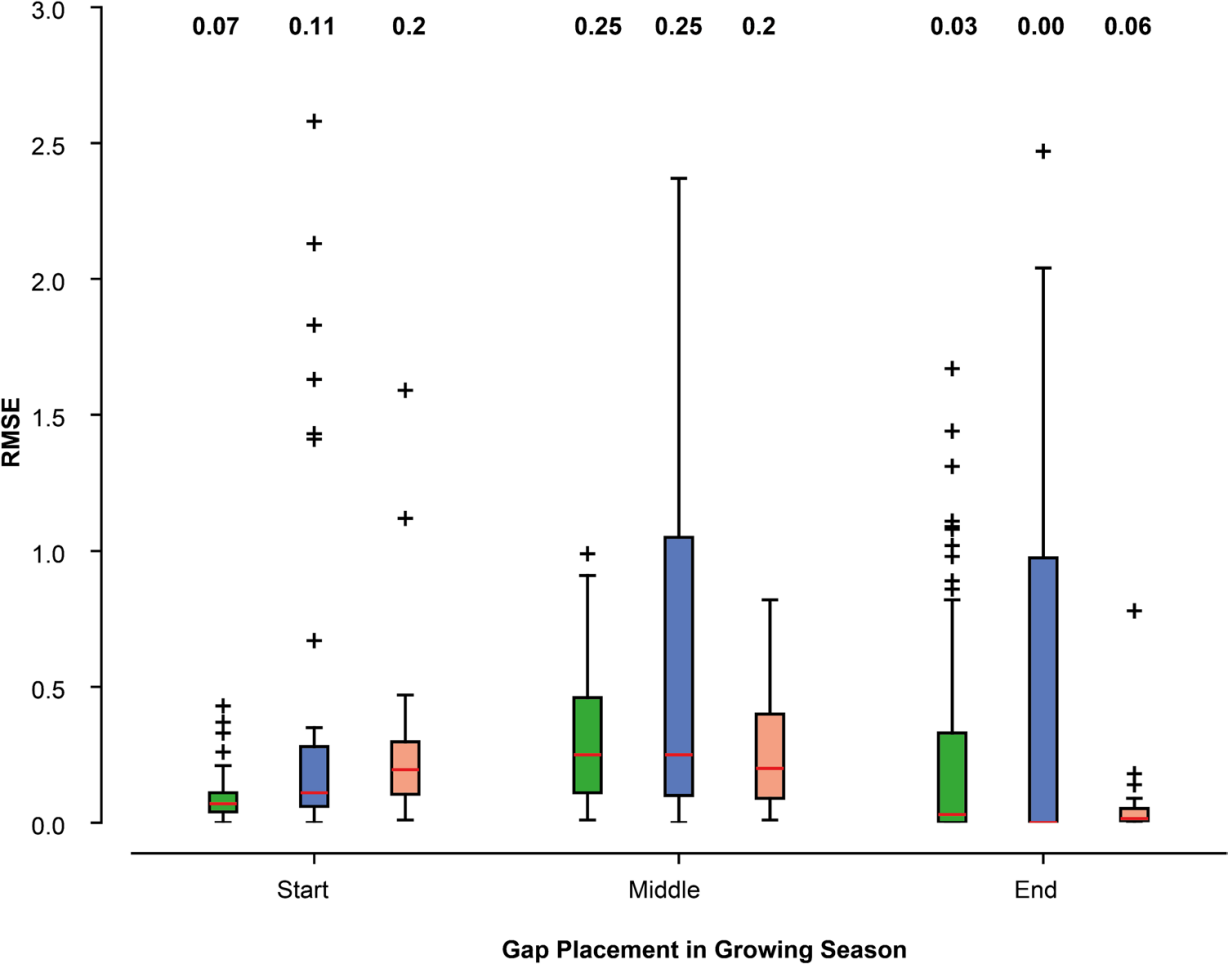
Comparison of RMSE values of the artificial gaps, when the gaps are filled with the individual tree models (green), spline interpolation (blue) or network interpolation (orange). On each boxplot, the red bar indicates the median, bottom and top edges indicate the 25^th^ and 75^th^ percentiles; the whiskers extend to all data points except outliers (drawn as “ + ”). The median RMSE values are given at the top of the figure. Highly significant (*p* > 0.01, Mann–Whitney *U* test) are the differences for the start of the growing season

The distribution of RMSE values for each seasonal gap demonstrated that network interpolation exhibited similar performance to the individual tree models during the middle of the growing season but had significantly higher RMSE values during the beginning of the growing season (Fig. 6). At the end of the growing season, the variance in RMSEs was considerably smaller for the network interpolation, although the number of gaps, where network interpolation could be applied, was limited (22.4%). For the start and middle of the season, it was possible to apply network interpolation to 16 and 26% of the artificially created gaps, respectively. Spline interpolation showed higher variance in RMSEs for the middle and end of the growing season. Significant differences (p < 0.01) were found between the individual tree and spline approach for the start of the growing season. For all approaches, the mid of the growing season means were higher than for the start and end.

In **Fig. S2**, the plane growth reconstruction for gaps at an urban example location for the year 2022 was highlighted in comparison to existing methods. The location and year were chosen as examples for the purpose of facilitating a more comprehensive comparison between methods, given that network interpolation could not be applied to all examples. Applied to the plane trees, the ML approach worked for all phases of the growing season with RMSE values ≤ 0.15 and the other approaches had higher RMSEs in most cases. The most striking visual observations were those, when spline interpolation predicted negative growth for the start of the season in 2022 and when the network interpolation reconstructed reduced growth for mid of growing season with an RMSE of 0.55.

The predictions for the maple tree at the same urban location (**Fig. S3**) revealed some shortcomings of the ML approach. The network interpolation approach made the most accurate predictions for the middle and end of the growing season. However, at the start of the growing season network interpolation did not capture the onset on the growing season, while the spline interpolation captured it best. Due to the way spline interpolation operates, this approach was unable to capture the characteristic stepwise growth in any gaps compared to the other approaches although RMSE values were generally low.

**Fig. 7:**
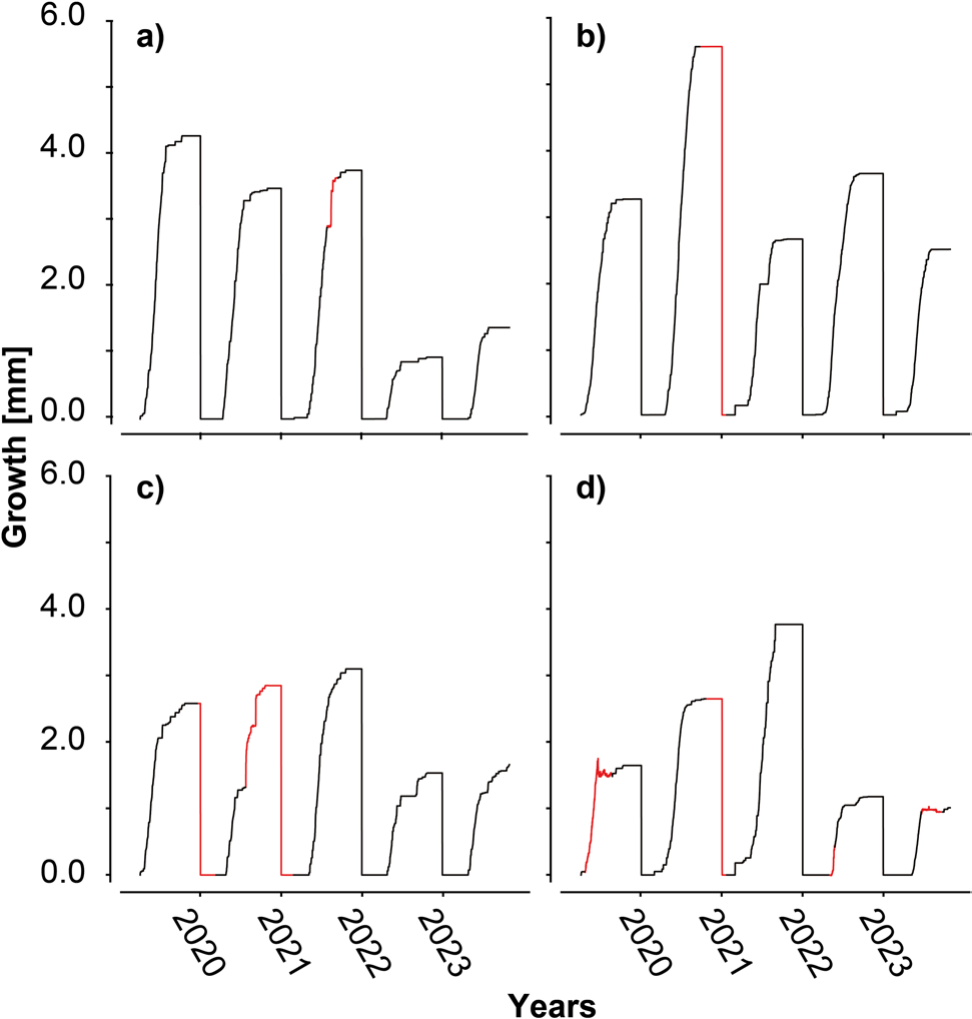
Reconstructed growth in actual existing gaps in the dendrometer data of this study: **a** urban maple, **b** urban plane, **c** non-urban maple and **d** non-urban plane

When the scheme is applied to true gaps of the datasets, the results clearly demonstrate the strength of the ML models to reconstruct long gaps in dendrometer data (Fig. 7). At visual inspection, the individual tree models were able to comprehensibly fill these gaps. It should be noted that the predictions for the non-urban plane tree (Fig. 7d) did not display reasonable growth values for the year 2019. The model predicted a reduction in growth values with increasing time. In such instances, we recommend that these erroneous predictions should be deleted and alternative methods like spline interpolation should be considered for these values. Users are encouraged to test various method on their capability to reconstruct data gaps. We recommend that any method used should be accompanied by a visual control of the reconstructed data.

The proposed ML method represents a complementary tool to existing methods as it enables the gap filling for individuals of a tree species. Consequently, the necessity for neighbouring trees with no gaps in the dendrometer data is negated. The findings indicate that individual tree models are capable of filling large data gaps. The application to actual gaps revealed that the models were capable of predicting reasonable values of growth even in gaps exceeding 30 days that span the transition between growth periods (start, middle, end) (Fig. 7). The low RMSE values (Fig. 6) suggest that the individual tree models can predict growth at the start and end of the growing season with high confidence. This allows, for example, a gapless analysis of the onset and cessation of tree growth in consecutive years. The ML method is not dependent on any climatic features. All features induced into the model are based on temporal variables and are hence available at any time. Features were controlled for multicollinearity before the application of any ML regression algorithm and dendrometer data has been carefully checked for quality. When the quality is poor, it is reflected in the generalization error and predictions made with these models are correspondingly unsatisfactory (Geiger et al. 2021; Briesch et al. 2022).

The application of this method, however, did not work for predicting raw dendrometer data and the models did not perform as good as the existing methods (**Fig. S4**). This is likely due to cyclical fluctuations of raw data due to stem growth, shrinkage and extension being more complex than continuously increasing stem growth data. Still, the introduced method offers a key advantage in handling long data gaps, especially when methods relying on sufficient data are not suitable (i.e. due to a lack of data from neighbouring trees). Future work should be undertaken to develop and test machine learning methods, which not only work for individual trees and independent from climatic variables but can also address positive and negative fluctuations in raw dendrometer data.

## Conclusion

We here introduced a novel method based on ML to fill dendrometer data gaps of individual trees exceeding 24h. Unlike previous approaches, our method is not dependent on climatic features or additional tree-growth data from neighbouring trees. Furthermore, we provided a comprehensive scheme that enables the replication of the method for other dendrometer data. The results showed that XGB-based models are capable of reconstructing tree-growth derived from dendrometer data at the start, middle and end of the growing season. Nonetheless, the comparison between methods revealed that no single universal method exists to fill long data gaps perfectly. The process of gap filling is complex and must be applied with caution. A visual evaluation of the predicted data is essential for any given case. Further research should explore the combination of methods and the functionality of the introduced ML approach on other tree species and environments.

## Supplementary Information

Below is the link to the electronic supplementary material.

Supplementary file1 (PDF 338 KB)

## Data Availability

Functions and code used to run an example, as well as example data are provided on GitHub as python codes (*.py and *.ipynb): https://github.com/ESKuhl/DM_GF_XGB.

## References

[CR1] Aryal S, Häusser M, Grießinger J, Fan Z, Bräuning A (2020) “dendRoAnalyst”: A tool for processing and analysing dendrometer data. Dendrochronologia 64:125772. 10.1016/j.dendro.2020.125772

[CR2] Barrao S, Serrano-Notivoli R, Cuadrat JM, Tejedor E, Saz Sánchez MA (2022) Characterization of the UHI in Zaragoza (Spain) using a quality-controlled hourly sensor-based urban climate network. Urban Climate 44:101207. 10.1016/j.uclim.2022.101207

[CR3] Beck C, Straub A, Breitner S, Cyrys J, Philipp A, Rathmann J, Schneider A, Wolf K, Jacobeit J (2018a) Air temperature characteristics of local climate zones in the Augsburg urban area (Bavaria, southern Germany) under varying synoptic conditions. Urban Clim 25(152):166. 10.1016/j.uclim.2018.04.007

[CR4] Beck HE, Zimmermann NE, McVicar TR, Vergopolan N, Berg A, Wood EF (2018b) Present and future Köppen-Geiger climate classification maps at 1-km resolution. Sci Data 5:180214. 10.1038/sdata.2018.214 30375988 10.1038/sdata.2018.214 PMC6207062

[CR5] Bischl B, Richter J, Becker M, Binder M, Lang M, Pielok T, Coors S, Thomas J, Ullmann T, Boulesteix A-L, Deng D, Lindauer M (2023) Hyperparameter optimization: Foundations, algorithms, best practices, and open challenges. Wires Data Mining Knowl Discov 13:1484. 10.1002/widm.1484

[CR6] Breiman L (2001) Random forests. Mach Learn 45:5–32. 10.1023/A:1010933404324

[CR7] Briesch M, Sobania D, Rothlauf F (2022) The randomness of input data spaces is an a priori predictor for generalization. In: Bergmann R, Malburg L, Rodermund SC, Timm IJ (eds) KI 2022: advances in artificial intelligence. Springer International Publishing, Cham, pp 17–30

[CR8] Cedro A, Nowak G (2006) Effects of climatic conditions on annual tree ring growth of the Platanus × hispanica ‘Acerifolia’ under urban conditions of Szczecin. Dendrobiology 55:11–17

[CR9] Chen T, Guestrin C (2016) XGBoost: a scalable tree boosting system. In: Proceedings of the 22nd ACM SIGKDD international conference on knowledge discovery and data mining. ACM, San Francisco California USA. pp 785–794

[CR10] Corell M, Girón IF, Galindo A, Torrecillas A, Torres-Sánchez R, Pérez-Pastor A, Moreno F, Moriana A (2014) Using band dendrometers in irrigation scheduling: influence of the location inside the tree and comparison with point dendrometer. Agric Water Manag 142:29–37. 10.1016/j.agwat.2014.04.005

[CR11] Deutscher Wetterdienst (2024) Mainz-Lerchenberg Station data. https://www.dwd.de/DE/leistungen/klimadatendeutschland/vielj_mittelwerte.html. Accessed 4 Apr 2024

[CR12] Dormann CF, Elith J, Bacher S, Buchmann C, Carl G, Carré G, Marquéz JRG, Gruber B, Lafourcade B, Leitão PJ, Münkemüller T, McClean C, Osborne PE, Reineking B, Schröder B, Skidmore AK, Zurell D, Lautenbach S (2013) Collinearity: a review of methods to deal with it and a simulation study evaluating their performance. Ecography 36:027–046. 10.1111/j.1600-0587.2012.07348.x

[CR13] Dulamsuren C, Coners H, Leuschner C, Hauck M (2023) Climatic control of high-resolution stem radius changes in a drought-limited southern boreal forest. Trees 37:797–810. 10.1007/s00468-022-02384-z

[CR15] Eischeid JK, Baker CB, Karl TR, Diaz HF (1995) The quality control of long-term climatological data using objective data analysis. J Appl Meteorol 34(12):2787–2795. 10.1175/1520-0450(1995)034%3c2787:TQCOLT%3e2.0.CO;2

[CR16] Fatima S, Hussain A, Amir SB, Ahmed SH, Aslam SMH (2023) XGBoost and random forest algorithms: an in depth analysis. Pak J Sci Res 3(1):26–31. 10.57041/pjosr.v3i1.946

[CR17] Fix E, Hodges JL (1951) Discriminatory analysis. Nonparametric discrimination consistency properties. USA Air Force School of Aviation Medicine, Randolph Field

[CR18] Friedman JH (2001) Greedy function approximation: a gradient boosting machine. Ann Statist 29(5):1189–1232. 10.1214/aos/1013203451

[CR19] Geiger RS, Cope D, Ip J, Lotosh M, Shah A, Weng J, Tang R (2021) “Garbage in, garbage out” revisited: what do machine learning application papers report about human-labeled training data? Quant Sci Stud. 10.1162/qss_a_00144

[CR20] Géron A (2019) Hands-on machine learning with Scikit-Learn, Keras & TensorFlow. Concepts, tools, and techniques to build intelligent systems, 2nd edn. O’Reilly, Sebastopol

[CR21] Google Satellite Image (2024) Google Maps. In: AeroWest Airbus, CNES/Airbus, GeoBasis-DE/BKH, GeoContent, Landsat/Copernicus, Maxar Technologies, Map data: GeoBasis-DE/BKG, Google

[CR22] Haeni M, Knüsel S, Wilhelm M, Peters RL, Zweifel R (2020) treenetproc—Clean, process and visualise dendrometer data. R package

[CR23] Head T, MechCoder GL, Shcherbatyi I (2018) scikit-optimize/scikit-optimize: v0. 5.2. Version v0 5

[CR24] Hoerl AE, Kennard RW (2000) Ridge regression: biased estimation for nonorthogonal problems. Technometrics 42(1):80–86. 10.1080/00401706.2000.10485983

[CR25] Iakovoglou V, Thompson J, Burras L, Kipper R (2001) Factors related to tree growth across urban-rural gradients in the Midwest, USA. Urban Ecosyst 5:71–85. 10.1023/A:1021829702654

[CR26] King G, Fonti P, Nievergelt D, Büntgen U, Frank D (2013) Climatic drivers of hourly to yearly tree radius variations along a 6 °C natural warming gradient. Agric Meteorol 168:36–46. 10.1016/j.agrformet.2012.08.002

[CR27] Knüsel S, Peters RL, Haeni M, Wilhelm M, Zweifel R (2021) Processing and extraction of seasonal tree physiological parameters from stem radius time series. Forests 12:765. 10.3390/f12060765

[CR28] Landeshauptstadt Mainz (2024) Jahresbericht Juli 2022–Juni 2023: Hauptamt Abteilung Öffentlichkeitsarbeit und Protokoll sowie städtische Ämter, Mainz

[CR29] Lindén J, Fonti P, Esper J (2016) Temporal variations in microclimate cooling induced by urban trees in Mainz, Germany. Urban Urban Green 20:198–209. 10.1016/j.ufug.2016.09.001

[CR30] Lukovic M, Zweifel R, Thiry G, Zhang C, Schubert M (2022) Reconstructing radial stem size changes of trees with machine learning. J R Soc Interface 19:20220349. 10.1098/rsif.2022.0349 36128707 10.1098/rsif.2022.0349 PMC9490331

[CR31] Lv H, Dermann A, Dermann F, Petridis Z, Köhler M, Saha S (2024) Comparable diameter resulted in larger leaf area and denser foliage in the park trees than in street trees: a study on Norway maples of Karlsruhe city, Germany. Heliyon 10:e23647. 10.1016/j.heliyon.2023.e23647 38187252 10.1016/j.heliyon.2023.e23647 PMC10767368

[CR32] Moser-Reischl A, Rahman MA, Pauleit S, Pretsch H, Rötzer T (2019) Growth patterns and effects of urban micro-climate on two physiologically contrasting urban tree species. Landsc Urban Plan 183:88–99. 10.1016/j.landurbplan.2018.11.004

[CR33] Pedregosa F, Varoquaux G, Gramfort A, Michel V, Thirion B, Grisel O, Blondel M, Prettenhofer P, Weiss R, Dubourg V, Vanderplas J, Passos A, Cournapeau D, Brucher M, Perrot M, Duchesnay E (2011) Scikit-learn: machine learning in python. J Mach Learn Res 12:2825–2830. 10.48550/arXiv.1201.0490

[CR34] R Core Team (2021) R: a language and environment for statistical computing. Austria, Vienna

[CR35] Rainio O, Teuho J, Klén R (2024) Evaluation metrics and statistical tests for machine learning. Sci Rep 14:6086. 10.1038/s41598-024-56706-x 38480847 10.1038/s41598-024-56706-x PMC10937649

[CR37] Salomón RL, Peters RL, Zweifel R, Sass-Klaassen UGW, Stegehuis AI, Smiljanic M, Poyatos R, Babst F, Cienciala E, Fonti P, Lerink BJW, Lindner M, Martinez-Vilalta J, Mencuccini M, Nabuurs G-J, van der Maaten E, von Arx G, Bär A, Akhmetzyanov L, Balanzategui D, Bellan M, Bendix J, Berveiller D, Blaženec M, Čada V, Carraro V, Cecchini S, Chan T, Conedera M, Delpierre N, Delzon S, Ditmarová Ľ, Dolezal J, Dufrêne E, Edvardsson J, Ehekircher S, Forner A, Frouz J, Ganthaler A, Gryc V, Güney A, Heinrich I, Hentschel R, Janda P, Ježík M, Kahle H-P, Knüsel S, Krejza J, Kuberski Ł, Kučera J, Lebourgeois F, Mikoláš M, Matula R, Mayr S, Oberhuber W, Obojes N, Osborne B, Paljakka T, Plichta R, Rabbel I, Rathgeber CBK, Salmon Y, Saunders M, Scharnweber T, Sitková Z, Stangler DF, Stereńczak K, Stojanović M, Střelcová K, Světlík J, Svoboda M, Tobin B, Trotsiuk V, Urban J, Valladares F, Vavrčík H, Vejpustková M, Walthert L, Wilmking M, Zin E, Zou J, Steppe K (2022) The 2018 European heatwave led to stem dehydration but not to consistent growth reductions in forests. Nat Commun 13(1):28. 10.1038/s41467-021-27579-9 35013178 10.1038/s41467-021-27579-9 PMC8748979

[CR38] Schwarz L, Sobania D, Rothlauf F (2024) On relevant features for the recurrence prediction of urothelial carcinoma of the bladder. Int J of Med Inform 186:105414. 10.1016/j.ijmedinf.2024.105414 38531255 10.1016/j.ijmedinf.2024.105414

[CR39] Ushey K, Allaire JJ, Tang Y (2024) Reticulate: interface to “python”. R package version 1.39.0. https://github.com/rstudio/reticulate, https://rstudio.github.io/reticulate/

[CR40] Ying X (2019) An overview of overfitting and its solutions. J Phys Conf Ser 1168:022022. 10.1088/1742-6596/1168/2/022022

[CR41] Zhang Y, Gao L, Deng Y, Huang Q, Yuan Y, Shi X (2024) Seasonal aridity regulates drivers and temporal variability of wood phenology: a meta-analysis of dendrometer monitoring data across the northern hemisphere. Dendrochronologia 85:126201. 10.1016/j.dendro.2024.126201

[CR42] Ziaco E, Biondi F (2018) Stem circadian phenology of four pine species in naturally contrasting climates from sky-island forests of the western USA. Forests 9(7):396. 10.3390/f9070396

[CR43] Zweifel R, Haeni M, Buchmann N, Eugster W (2016) Are trees able to grow in periods of stem shrinkage? New Phytol 211:839–849. 10.1111/nph.13995 27189708 10.1111/nph.13995

