# Supplementary material for: A machine learning approach to fill gaps in dendrometer data
Source: Trees (Berl West). 2024 Oct 15;38(6):1557–67. doi: 10.1007/s00468-024-02573-y (PMC11584462; doi:10.1007/s00468-024-02573-y)
Supplement: Supplementary file 1 — Supplementary file1 (PDF 338 KB) [file 468_2024_2573_MOESM1_ESM.pdf]

Submitted to Trees

A machine learning approach to fill daps in dendrometer data

Eileen Kuhl (0000-0002-1246-6030)[1], Emanuele Ziaco (0000-0002-0459-6927)[1], Jan Esper (0000-0003-3919-014X)[1,2], Oliver Konter[1], Edurne Martinez del Castillo (0000-0003-1542-2698)[1]

[1] Department of Geography, Johannes Gutenberg University, Mainz, Germany

[2]Global Change Research Centre (CzechGlobe, Brno, Czech Republic

Corresponding author: Eileen Kuhl (eikuhl@uni-mainz.de, Johann-Joachim-Becher Weg 32, 55128 Mainz, Germany, Orcid: 0000-0002-1246-6030

Table S1 Regression algorithms tested in this approach

#	Regression Algorithm	Type
1	Linear	Linear
2	Ridge	Linear
3	Lasso	Linear
4	Random Forest (RF)	Nonlinear
5	k-Nearest-Neighbor (kNN)	Nonlinear
6	Support Vector Machine (SVM)	Linear
7	Extreme Gradient Boosting (XGB)	Nonlinear
8	Partial Least Square (PLS)	Linear

Table S2 Performance results (RMSE and adjusted R^2) of eight algorithms trained on datasets #3-6 (Tabl. 1) to predicting growth. Performance measures were estimated using repeated k-fold cross validation (k = 10, repeats = 10). Performance values shown here equal the mean of the 100 runs

**Urban Maple**			**Urban Plane**			**Non-urban Maple**			**Non-urban Plane**		
**Model**	**RMSE**	**adjR²**	**Model**	**RMSE**	**adjR²**	**Model**	**RMSE**	**adjR²**	**Model**	**RMSE**	**adjR²**
RF	0.00	1.00	RF	0.00	1.00	RF	0.00	1.00	RF	0.00	1.00
XGB	0.01	1.00	XGB	0.02	1.00	XGB	0.01	1.00	XGB	0.01	1.00
kNN	0.10	0.99	kNN	0.13	0.99	kNN	0.04	1.00	kNN	0.06	1.00
SVM	0.49	0.8	SVM	0.92	0.71	SVM	0.33	0.82	SVM	0.51	0.82
Ridge	0.74	0.54	Ridge	0.96	0.69	Ridge	0.47	0.63	Ridge	0.64	0.73
Linear	0.74	0.54	Linear	0.96	0.69	Linear	0.47	0.63	Linear	0.64	0.73
Lasso	0.74	0.54	Lasso	0.96	0.69	Lasso	0.47	0.63	Lasso	0.64	0.73
PLS	0.74	0.54	PLS	0.96	0.69	PLS	0.52	0.56	PLS	0.64	0.72

Table S3 Ranked performance results from hyperparameter tuning to fit different algorithms to the datasets #3-6 (Tabl. 1). Best RMSE scores for all four datasets are shown in reference to growth in millimetres. Param. denotes to the best hyperparameters found via Bayesian Optimization Search and 10-fold cross validation

**Position**	**Urbane Maple**			**Urban Plane**			**Non-urban Maple**			**Non-urban Plane**		
	**Model**	**RMSE**	**Param.**	**Model**	**RMSE**	**Param.**	**Model**	**RMSE**	**Param.**	**Model**	**RMSE**	**Param.**
1	**XGB**	0.01	colsample_bytree= 1, eta= 0.16, gamma= 10, max_depth= 15, min_child_weight= 7, subsample= 0.9, early_stopping_rounds=10	**XGB**	0.01	colsample_bytree= 1, eta= 0.1, gamma= 30, max_depth= 13, min_child_weight= 8, subsample= 0.9, early_stopping_rounds=10	**XGB**	0.00	colsample_bytree= 1, eta= 0.16, gamma= 50, max_depth= 13, min_child_weight= 6, subsample= 0.9, early_stopping_rounds=10	**XGB**	0.01	colsample_bytree= 1, eta= 0.1, gamma= 30, max_depth= 15, min_child_weight= 3, subsample= 0.6, early_stopping_rounds=10
2	**RF**	0.02	bootstrap= True, max_depth= 86, max_features= 1.0, max_leaf_nodes = 95, n_estimators= 116, random_state= 42	**RF**	0.07	bootstrap= True, max_depth= 66, max_features= 1.0, max_leaf_nodes= 95, n_estimators= 186, random_state= 42	**RF**	0.02	bootstrap= True, max_depth= 75, max_features= 1.0, max_leaf_nodes = 95, n_estimators= 170, random_state= 42	**RF**	0.05	bootstrap= True, max_depth= 46, max_features= 1.0, max_leaf_nodes= 98, n_estimators= 196, random_state= 42
3	**kNN**	0.08	algorithm= 'ball_tree', leaf_size= 31, metric= 'manhattan', n_neighbors= 5, p= 2, weights= 'distance'	**kNN**	0.11	algorithm= 'brute', leaf_size= 11, metric= 'manhattan', n_neighbors= 5, p= 1, weights= 'distance'	**kNN**	0.04	algorithm= 'kd_tree', leaf_size= 66, metric= 'euclidean', n_neighbors= 5, p= 2, weights= 'distance'	**kNN**	0.06	algorithm= 'brute', leaf_size= 11, metric= 'euclidean', n_neighbors= 7, p= 2, weights= 'distance'
4	**Ridge**	0.74	alpha= 1.0	**Ridge**	0.96	alpha= 1.0	**Ridge**	0.47	alpha= 0.4	**Ridge**	0.64	alpha= 1.0

Table S4 Test set RMSE values of the models based on the datasets #1-6 and the four algorithms extreme gradient boosting (XGB), k-nearest neighbor (kNN), random forest (RF) and ridge regression after hyperparameter tuning.

#	X	XGB	RF	kNN	Ridge
**1**	Maple	0.01	0.05	0.07	0.78
**2**	Plane	0.01	0.10	0.10	0.94
**3**	Urban Maple	0.01	0.02	0.09	0.74
**4**	Urban Plane	0.01	0.07	0.12	0.95
**5**	Non-Urban Maple	0.00	0.02	0.04	0.47
**6**	Non-Urban Plane	0.01	0.05	0.06	0.64

Table S5 Test set RMSE mean values and standard deviation (in brackets) of the models based on the datasets #3-8 and the four algorithms extreme gradient boosting (XGB), k-nearest neighbor (kNN), random forest (RF) and ridge regression without hyperparameter tuning and VPD

#	X	XGB	RF	kNN	Ridge
**3**	Urban Maple	1.03 (0.01)	1.36 (0.01)	1.15 (0.01)	1.14 (0.01)
**4**	Urban Plane	0.69 (0.01)	0.92 (0.01)	0.78 (0.01)	1.55 (0.01)
**5**	Non-Urban Maple	0.77 (0.01)	1.00 (0.01)	0.88 (0.01)	1.02 (0.01)
**6**	Non-Urban Plane	0.61 (0.01)	0.80 (0.01)	0.70 (0.01)	1.05 (0.00)
**7**	Urban 1 Maple	0.02 (0.01)	0.00 (0.00)	0.03 (0.00)	0.27 (0.00)
**8**	Urban 1 Plane	0.07 (0.08)	0.00 (0.00)	0.18 (0.01)	1.20 (0.01)
**9**	Urban 2 Maple	0.13 (0.10)	0.00 (0.00)	0.19 (0.01)	1.29 (0.01)
**10**	Urban 2 Plane	0.01 (0.00)	0.00 (0.00)	0.25 (0.01)	2.00 (0.01)
**11**	Urban 3 Maple	0.01 (0.00)	0.00 (0.00)	0.06 (0.01)	0.40 (0.00)
**12**	Urban 3 Plane	0.04 (0.07)	0.00 (0.00)	0.16 (0.01)	1.25 (0.02)
**13**	Non-urban 1 Maple	0.05 (0.09)	0.00 (0.00)	0.10 (0.00)	0.87 (0.01)
**14**	Non-urban 1 Plane	0.11 (0.09)	0.00 (0.00)	0.17 (0.00)	1.16 (0.01)
**15**	Non-urban 2 Maple	0.03 (0.03)	0.00 (0.00)	0.05 (0.00)	0.36 (0.00)
**16**	Non-urban 2 Plane	0.06 (0.05)	0.00 (0.00)	0.10 (0.00)	0.66 (0.01)
**17**	Non-urban 3 Maple	0.01 (0.01)	0.00 (0.00)	0.03 (0.00)	0.25 (0.00)
**18**	Non-urban 3 Plane	0.01 (0.00)	0.00 (0.00)	0.14 (0.01)	1.16 (0.02)

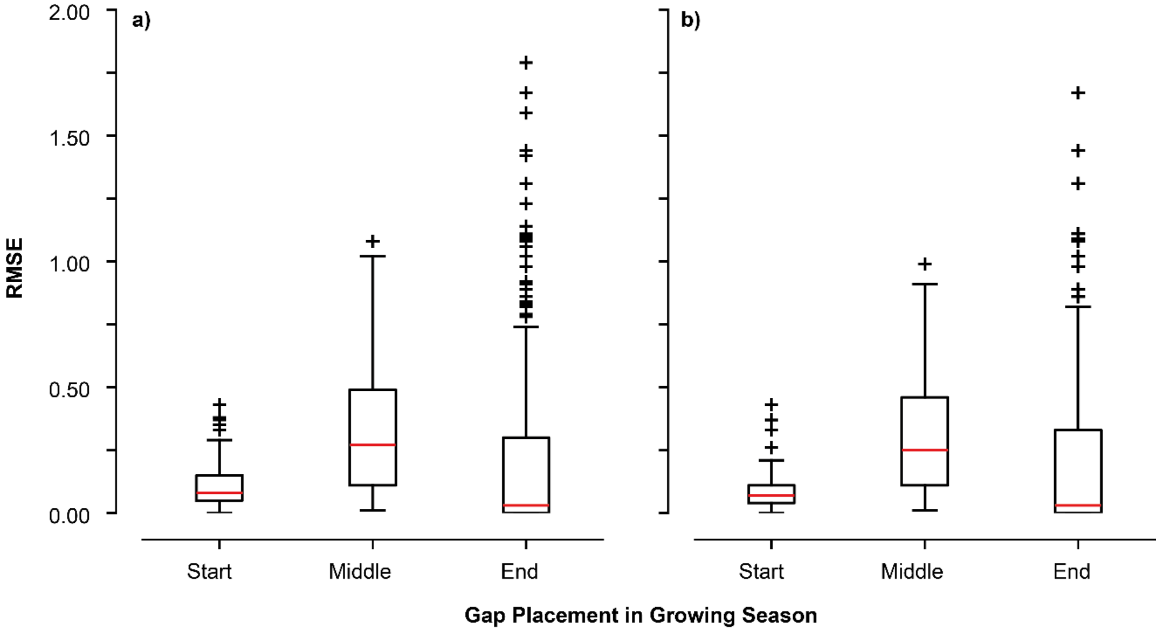

Figure S1 Comparison of RMSE values of the artificial gaps, when the models are based on **a)** random forest and **b)** extreme gradient boosting. On each boxplot, the red bar indicates the median, bottom and top edges indicate the 25th and 75th percentiles; the whiskers extend to all data points except outliers (drawn as “+”). No significant differences could be found for the middle and end of the growing seasons ($p > 0.01$, Mann-Whitney-U test)

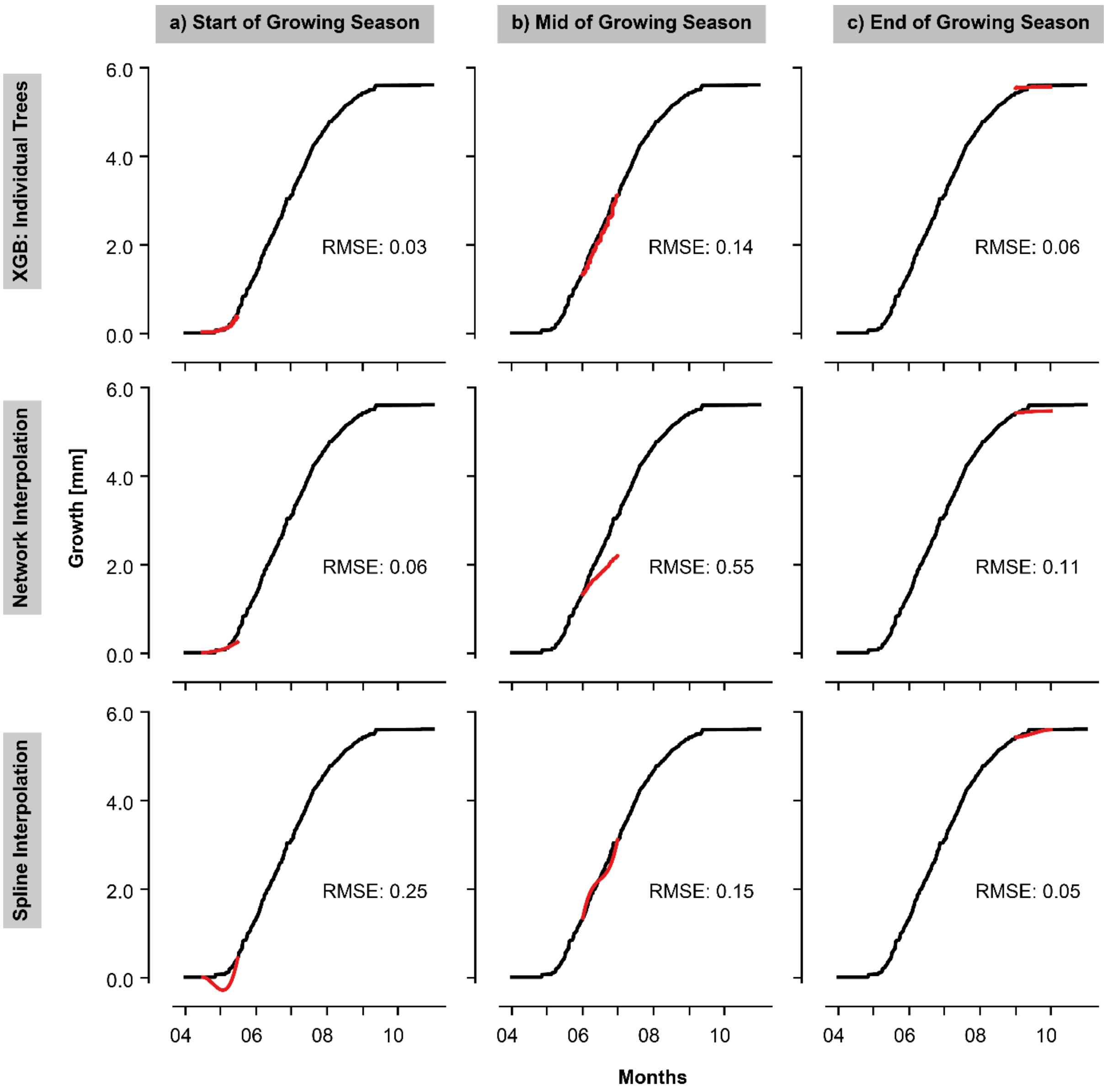

Figure S2 Results of different approaches for predicting growth of the maple tree at an urban location of 2022 CE fitted to the algorithm Extreme Gradient Boosting (XGB) for different seasons (**a-c)**). Top row shows the results of the individual Urban Maple models. Middle and bottom row present the comparison to the network interpolation by Aryal et al. (2020) and the classic spline interpolation

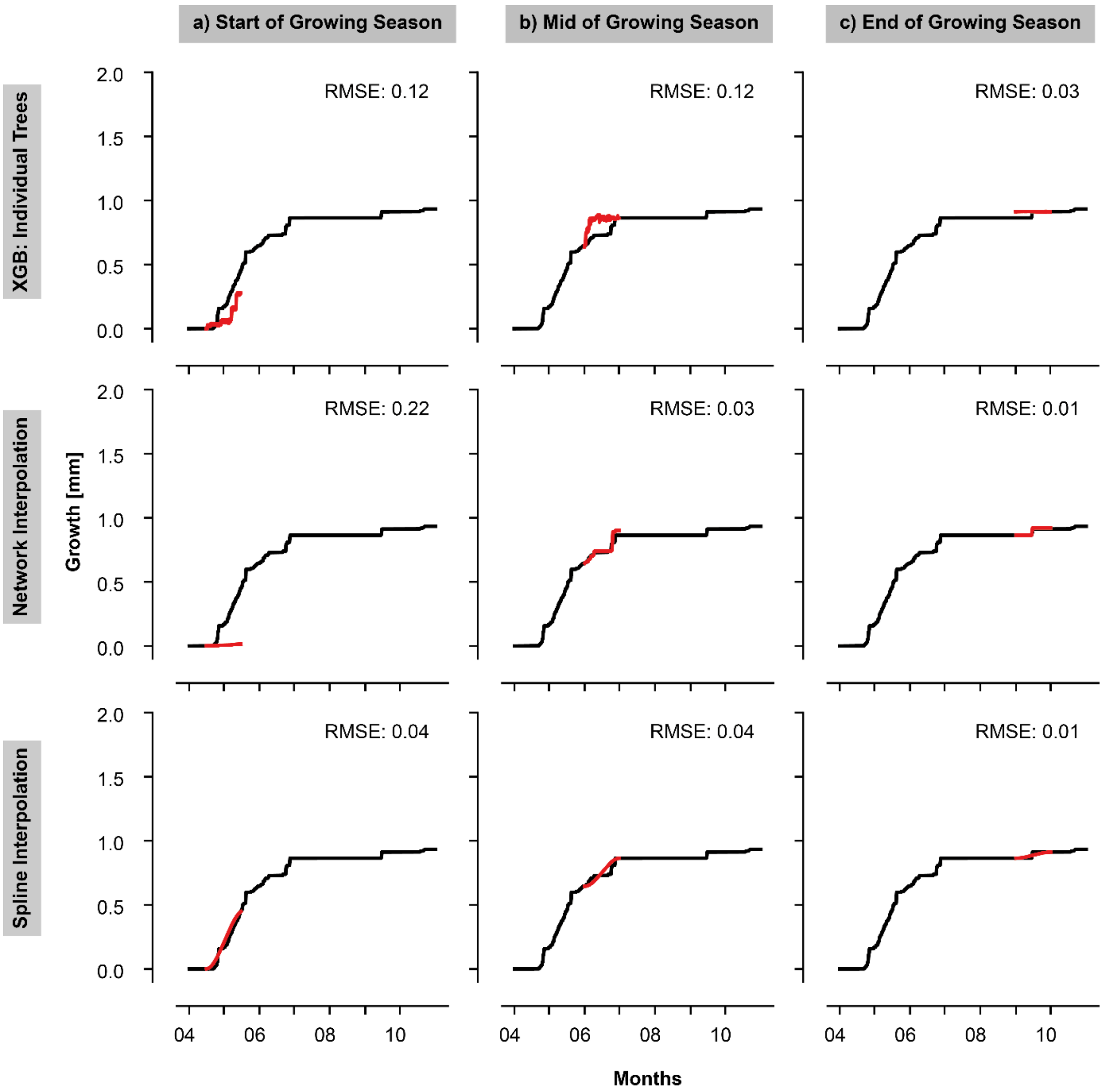

Figure S3 Results of different approaches for predicting growth of the maple tree at an urban location in 2022 CE fitted to the algorithm Extreme Gradient Boosting (XGB) for different seasons (**a-c)**). Top row shows the results of the individual Urban Maple models. Middle and bottom row present the comparison to the network interpolation by Aryal et al. (2020) and the classic spline interpolation

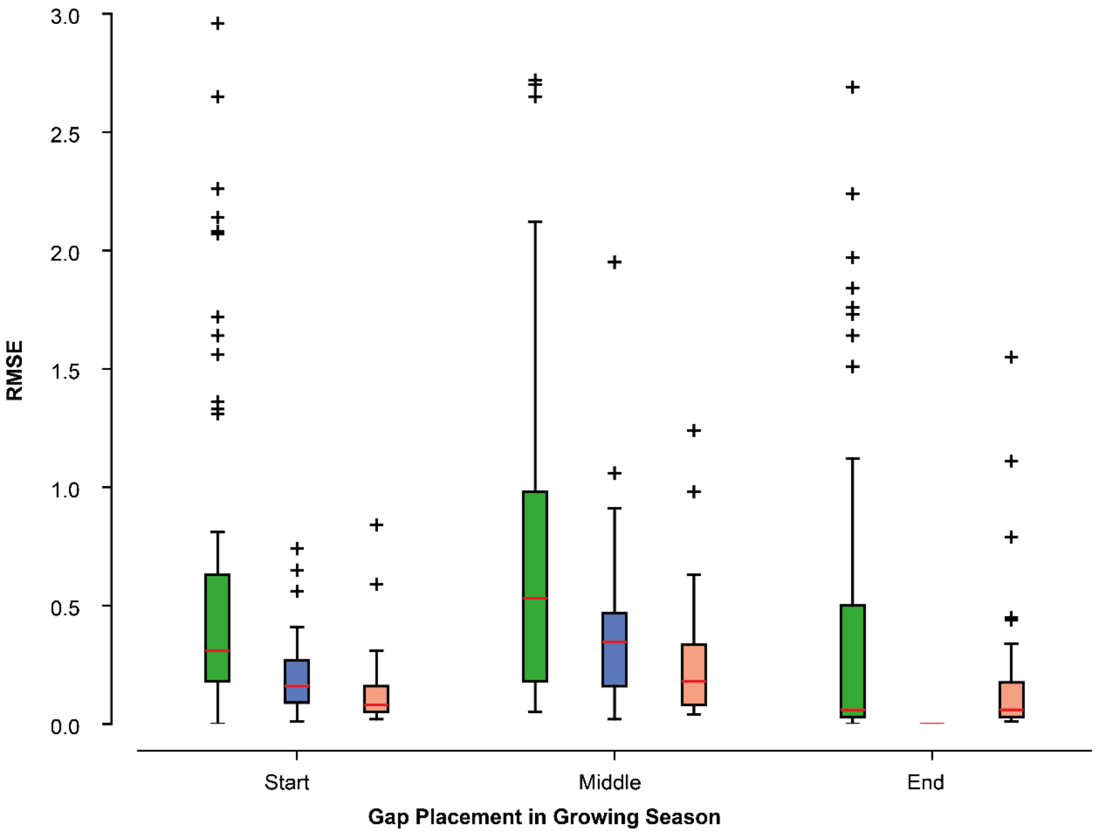

Figure S4 Comparison of RMSE values of the artificial gaps from raw dendrometer data, when the gaps are filed with the individual tree models (green), spline interpolation (blue) or network interpolation (orange). On each boxplot, the red bar indicates the median, bottom and top edges indicate the 25th and 75th percentiles; the whiskers extend to all data points except outliers (drawn as "+").
